# A universal platform for building molecular logic circuits based on a reconfigurable three-dimensional DNA nanostructure†
†Electronic supplementary information (ESI) available: All DNA sequences used in this study; detailed descriptions of the native PAGE experiments; the experiments investigating the thermal stability; the experiments of switching the DNA prism between the closed and opened states; the serum stability of the prism; and the experiments of logic gate operation in a biological matrix. See DOI: 10.1039/c5sc00371g


**DOI:** 10.1039/c5sc00371g

**Published:** 2015-04-08

**Authors:** Kaiyu He, Yong Li, Binbin Xiang, Peng Zhao, Yufang Hu, Yan Huang, Wang Li, Zhou Nie, Shouzhuo Yao

**Affiliations:** a State Key Laboratory of Chemo/Biosensing and Chemometrics , College of Chemistry and Chemical Engineering , Hunan University , Changsha , 410082 , P. R. China . Email: niezhou.hnu@gmail.com ; Fax: +86-731-88821848 ; Tel: +86-731-88821626

## Abstract

Molecular logic gates are capable of performing various logic tasks for biomarker detection, disease diagnostics and therapy, and controlling biological progress. Herein, we integrated multiple components of a logic device into a single DNA 3D nano-assembly with a triangular prism structure. Compared with the separate construction of each component in previously reported DNA logic gate systems, such an integrated design strategy made the 3D DNA nanoprism universal for logic gates, it can be reconfigured to execute diverse logic operations. Binary basic logic gates (OR, AND, INHIBIT and XOR), combinatorial gates (INHIBIT–OR), and multi-valued logic gates (ternary INHIBIT gate) were readily achieved by taking this DNA nanoprism as a universal platform. Moreover, a logic gate system for identification of even numbers and odd numbers from natural numbers was established successfully by employing only this single DNA nanoprism and four short single-stranded DNA. The universality of this nanoprism greatly simplified the design of DNA logic gate system. Additionally, this nanoprism was able to perform logic operation steadily in a biological matrix, indicating that this box-like DNA nanostructure applies to logic gates in a complicated environment. This study provided a unique opportunity to design versatile 3D DNA nanostructure-based intelligent nanodevices, which show great potential in biocomputing, multi-parameter sensing, and intelligent disease diagnostics and therapy.

## Abstract 2

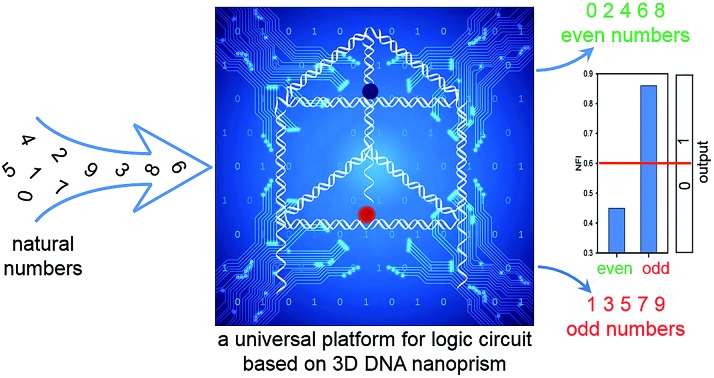
Integrating multiple components of a logic device into a 3D DNA nanoprism provides a universal platform for constructing diverse logic gates.

## Introduction

Modern computers use silicon-based logic gates to convert input signals into a defined output for information processing. Similarly, molecular logic gates^1^–^3^ use molecules as inputs to perform a wide variety of logic tasks, and show great potential in many life science applications, such as biomarker detection,^4^–^6^ disease diagnostics and therapy,^7^–^9^ controlling biological progress,^10^ and promoting the cognition of life phenomena for humans.^2^,^11^ For information technology, computational problems arising from miniaturization limitations and operation speeds associated with the top-down approach could potentially be circumvented using molecular circuits built in a bottom-up manner.^12^ Some computationally intractable problems have been solved experimentally by exploiting the parallel reaction of large numbers of molecules for highly parallel computing.^13^–^15^

The DNA molecule, which encodes the complex forms of life on earth, is one of the most attractive building blocks for developing molecular logic gates due to its unique features, such as sequence-specific hybridization based on precise base-pairing, as well as the catalytic activity (*e.g.*, DNAzymes) and recognition capability (*e.g.*, aptamers) of specific DNA sequences. In addition, the synthesis and site-specific modification of DNA is facile. Thus, DNA logic gates have been actively pursued and ingenious logic gate systems have been created.^4^–^8^,^13^–^21^ Typically, a logic device consists of three components: sensors that receive input information; processors that interpret and respond to this information; and actuators that carry the response out.^3^ In most existing DNA logic gate systems, the three components were constructed separately based on one-dimensional (1D) or two-dimensional (2D) DNA nanostructures, such as simple DNA duplex, hairpin structure, DNA-junction architectures, *etc.* Such strategies result in a logic device with a decentralized structure and limited types of logic gates that a single logic device can accomplish, hindering the integration of elementary logic gates into higher-level circuits. Considering possible biological applications, most of these DNA logic gate systems are not competent enough to carry out logic tasks in biological matrices due to their lack of resistance to enzymatic degradation. Therefore, there is in urgent need to develop a universal platform to build logic gates for highly integrated circuits, which is able to execute logic tasks in both ideal experimental conditions and complicated environments.

In recent years, a diverse variety of addressable and controllable 3D nanostructures have been created by using DNA nanotechnology.^22^–^27^ Owing to the predictable and programmable interactions of DNA with natural or synthetic molecules, 3D DNA nanostructures have tremendous potential for 3D organization of functional components, serving as molds to cast inorganic nanostructures with arbitrarily prescribed 3D shapes, encapsulating and releasing drugs, proteins and nanomaterials, and allowing the exploration of new properties for encapsulated guest molecules.^28^–^31^ Several works have recently demonstrated that compact 3D DNA nanostructures facilitate their intracellular uptake and confer even greater stability to their component strands under physiological conditions,^32^–^34^ which guarantee the delivery of multiple functional components to enter a cell with an exact ratio and the synergetic execution of their functions *in vivo*. Hence, the introduction of 3D DNA nanostructures into DNA logic circuits might provide a generic platform for designing intelligent systems that potentially function *in vivo* to analyse logically multiple items of information and then respond appropriately, such as DNA nanocarriers for on-demand target delivery in a stimuli-responsive fashion. In turn, the development of such strategies to reconfigure logically 3D DNA nanostructures through clever stimuli, can also promote the full realization of their potential for more user-specified functional materials. However, the applications of 3D DNA nanostructures to construct molecular logic gates are scarce. So far, only two pioneering works have been reported: in one Douglas *et al.* designed a DNA origami-based nanobarrel with several different logical AND gates to control the release of payloads.^35^ In the other Fan *et al.* constructed logic gates by using a series of tetrahedral DNA nanostructures that were responsive to protons, Hg^2+^, ATP and DNA strands.^36^

Herein, we have assembled the multiple components of a logic device into a 3D DNA triangular nanoprism with an integrated design strategy. The box-like nanoprism contains three single-stranded DNA segments elongated from its three vertical edges, which serve as sensors to receive different input information and open the box responsively in various ways for logical operation. As there was a fluorophore and a quencher labeled in the upper and lower lids of the box, respectively, the structural reconfigurations responding to external stimuli were reflected by different fluorescence intensities, which served as the output signals. To execute logic gate operation, we employed single-stranded DNA as inputs to activate this self-assembled nanoprism *via* a toehold-mediated DNA strand displacement reaction (SDR).^37^ Our integrated design endowed the DNA nanoprism with versatility and the single DNA nanoprism can serve as a universal platform for various logic operations, such as binary basic logic gates (OR, AND, INHIBIT and XOR), combinatorial gates (INHIBIT–OR) and multi-valued logic gates (ternary INHIBIT gate). Moreover, a logic gate system for identification of even numbers and odd numbers from natural numbers was successfully established by employing only this single prism and four single-stranded DNA. In addition, we demonstrated that this 3D DNA nanoprism was able to execute stably logic operation in a biological matrix.

## Results and discussion

### Fabrication of the 3D DNA nano-assembly with triangular prism structure

**Scheme 1:**
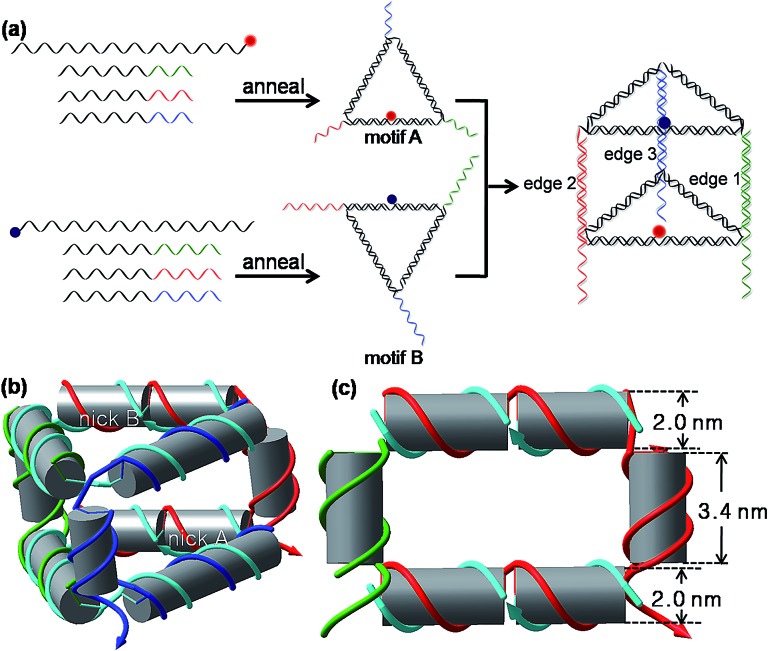
(a) Schematic representation of the self-assembly of the DNA triangular prism. (b) The structural model of the prism. Two nicks (nick A and nick B, break in the DNA backbone), where the 5′ and 3′ terminals of strand L meet, were included in the middle of the L/A2 and L/B2 double helical edges, respectively. (c) The geometric model of part of the prism for estimating the distance *r* between nick A and nick B (the two models were produced by the computer aided design program *NanoEngineer-1* (version 1.1.1, Nanorex INC.)).

As shown in Scheme 1(a) and S1,† the 3D DNA nanoprism is assembled modularly using a DNA-economic strategy. A long central strand L and three short strands A1, A2, and A3 first assemble into an equilateral triangular face motif A based on sequence-specific complementarity, and each side of the motif A is two helical turns (21 base pairs (bp)) long. The 10 nucleotide (nt) unpaired segments of A1, A2, and A3 serve as the single-stranded DNA (ssDNA) tails at each vertex of motif A. The L strand is cyclized by hybridization with A2, thus its 5′ and 3′ ends meet in the middle of the L/A2 side, forming a nick in the DNA backbone. A similar design strategy is applied to the assembly of the equilateral triangular face motif B by an identical L and different set of short strands (B1, B2, and B3) containing 16-nt long tails. The construction of the 3D nanoprism is achieved by hetero-dimerization of motif A and motif B *via* the hybridization of those tails. Each vertical side of the nanoprism composed by the hybrid of A and B strands (A1–B1, A2–B2, or A3–B3) is one helical turn (10 nt) long. The 6 nt ssDNA protrusive parts of the B strand (B1, B2 and B3) at each vertical side function as toehold domains to sense the input DNA in the following studies. Thus, the self-assembled prism has vertical sides with length about 3.40 nm (10 bp), and equilateral triangle bases with side lengths about 7.14 nm (21 bp); the inner volume is approximately 75 nm^3^. A structural model of the prism is shown in Scheme 1(b). Native polyacrylamide gel electrophoresis (PAGE) was employed to verify the self-assembly of the prism (Fig. S1, ESI†). All the well-formed complexes migrated in the gel as expected. For each of the triangular motifs, a major band appeared (lane 6 and 9), which can be easily identified from the control groups. When the two triangular motifs were mixed together, a new dominant band appeared (lane 10), corresponding to the heterodimer of the two triangular motifs, namely the triangular prism. The experimental results also indicated that the yield of the prism was good (lane 10). Therefore, the raw nanoprism was used directly without further purification in the subsequent experiments in order to simplify the preparation, reduce the cost, and improve the practicality of the nanoprism-based logic system.

For fluorescence measurement, a fluorophore ROX and a quencher BHQ-2 were labeled at nick A and nick B, respectively, by terminal-labeling strand L. Since the fluorescence resonance energy transfer (FRET) efficiency depends on the distance between donor–acceptor pair and the relative orientation of the donor and acceptor transition dipoles, the choice of terminals of L strands for labeling was optimized for the maximal FRET efficiency and minimum background fluorescence of the intact prism (Fig. S2, ESI†). As shown in Fig. S2,† a minimum background fluorescence (the self-assembled prism fluoresces at 37.5% of the unquenched fluorescence) was obtained when the fluorophore ROX was labeled at the 3′ terminal of strand L in motif A and the quencher BHQ-2 was labeled at the 5′ terminal of strand L in motif B. This optimal result was adopted in the subsequent studies. According to their respective fluorescence and absorption spectra, the FRET calculated distance *r* between the fluorophore and the quencher within the prism was 5.5 nm, while the estimated distance *r* between the two nicks in this prism with a 10 bp vertical edge was estimated to be 5.4 nm from the geometric model (Scheme 1(c) and Fig. S3, ESI†). These two values are well consistent with each other, confirming the self-assembly of the prism once again. The thermal stability of the prism structure was investigated *via* the fluorescence signal change of the DNA nanoprism in response to the continuous increasing temperature (Fig. S4, ESI†). As the temperature increases, the fluorescence intensity starts a sharp increase at 43 °C and then reaches a maximum at 57 °C. The temperature at which the rise in the fluorescence intensity was half maximal is about 50 °C. The melting curve indicates that the prism was stable enough at room temperature or physiological temperature (37 °C).

### Reconfigurations of the DNA triangular prism

**Fig. 1:**
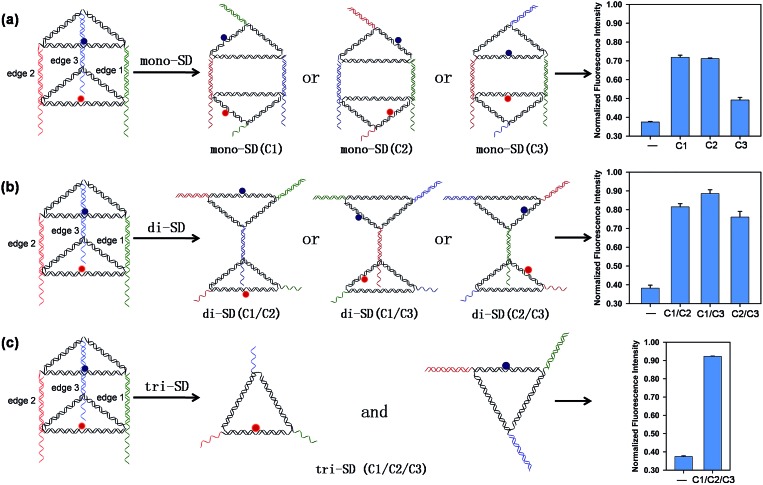
Reconfigurations of the DNA triangular prism *via* the toehold-mediated DNA strand displacement reaction (SDR) and the normalized fluorescence intensities of nanostructures derived from reconfigurations after different combinations of C1, C2, and C3 (as shown in parenthesis) were introduced to initiate SDRs on the corresponding vertical edge 1, edge 2 and edge 3, respectively. SDRs occur on one (mono-SD, (a)), two (di-SD, (b)), and all (tri-SD, (c)) of the three edges, respectively. The fluorescence intensity of ROX was measured at 603 nm.

Three short single-stranded DNA of C1, C2, and C3, which are complementary to the 16 nt tail of B1, B2, and B3, respectively, were introduced to initiate DNA strand displacement on the corresponding edges, inducing reconfigurations of the prism. Reconfiguring the prism will alter the distance between the donor and the acceptor, thus affecting the relative orientation of the donor and acceptor transition dipoles, resulting in different FRET efficiency, leading to the fluorescence variation of ROX. Fig. 1 shows the results when different combinations of C1, C2, and C3 were applied. The upper and lower triangular lids of the prism were opened and similar reconfigured structures (mono-SD(C1), mono-SD(C2) and mono-SD(C3)) were obtained when C1 or C2 or C3 was input separately. In structures of mono-SD(C1) and mono-SD(C2), the distance between ROX and BHQ-2 increased as the prism was opened, increasing obviously the fluorescence intensity of ROX. Meanwhile, in structure mono-SD(C3) the relative position of ROX and BHQ-2 was not affected and a tiny increase of fluorescence intensity was observed. When two of the three ssDNA were combined to deform the prism, three reconfigured nanostructures of two triangular motifs linked by a 10 bp duplex (di-SD(C1/C2), di-SD(C1/C3) and di-SD(C2/C3)) were acquired, and the fluorescence intensity was enhanced considerably. The simultaneous input of C1, C2 and C3 caused the prism to deconstruct into two triangular motifs (tri-SD(C1/C2/C3)), accompanied by an almost full fluorescence intensity recovery. Also, native PAGE was applied to confirm these reconfigurations, and all the nanostructures resulted from reconfigurations that migrated reasonably in the gel (Fig. S5, ESI†). These interesting phenomena laid the foundation for creating molecular logic gates by using the DNA nanoprism as a universal work unit. Then, a series of binary logic gates were constructed by employing single-stranded DNA as input. The presence and absence of the single-stranded DNA were assigned as the respective inputs of [1] and [0], and the normalized fluorescence intensity (the fluorescence intensity at 603 nm of totally unquenched ROX in prism without BHQ-2 was normalized to 1.0 for normalization) value of 0.6 (NFI_603_ = 0.6) was defined as the threshold value for all the binary logic gates.

### Construction of binary basic logic gates

**Fig. 2:**
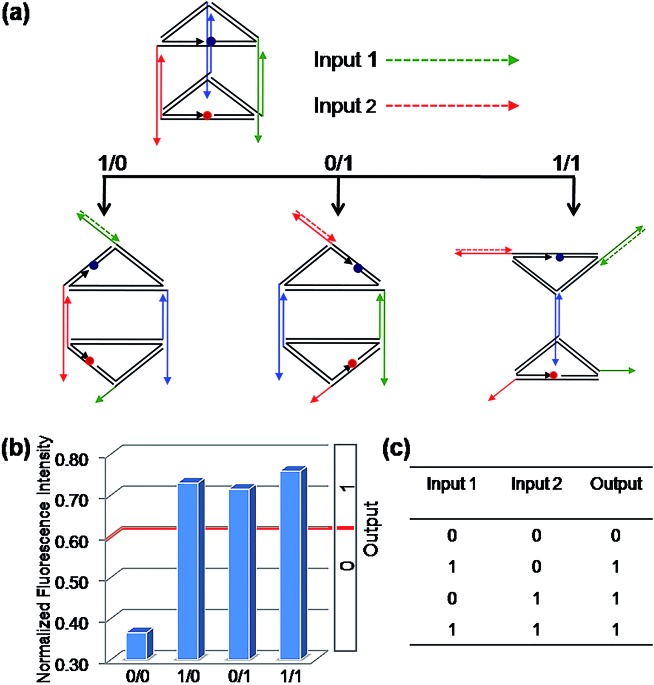
The binary “OR” logic gate. (a) Diagram of the operational design of the “OR” gate. Solid and dashed lines with the same color are complementary to each other. (b) The NFI_603_ of ROX. (c) The truth table of the “OR” logic gate.

Firstly, the binary OR logic gate was easily achieved by applying C1 and C2 as Input 1 and Input 2, respectively (Fig. 2). Both of the two single-stranded DNA can open the prism: C1 initiates a DNA strand displacement on edge 1, generating the structure mono-SD(C1). Similarly, C2 is capable of conducting a strand displacement on edge 2, producing the structure mono-SD(C2). As a result, either of the two inputs, C1 or C2, as well as the two inputs together (producing structure di-SD(C1/C2)), can reconfigure the prism, leading to an increase in the distance between ROX and BHQ-2, with concomitant NFI_603_ values higher than the threshold value 0.6. Thus, when the input state was (0/0), the output was [0], while other input states (1/0), (0/1) and (1/1) displayed the true output [1]. These results are in accordance with the proper execution of the binary OR logic gate. Then, the binary INHIBIT logic gate was achieved by employing C1 and its complementary strand C1′ (Fig. S6†) or C2 and its complementary strand C2′ (Fig. S7, ESI†) as the respective Input 1 and Input 2, respectively. Since C1′ or C2′ pair with their respective complementary strand to prohibit reconfiguration of the prism, the true output [1] was obtained only when C1 (or C2) was introduced and the input state was (1/0), while the other input states (0/0), (0/1), (1/1) generated the false output [0].

**Fig. 3:**
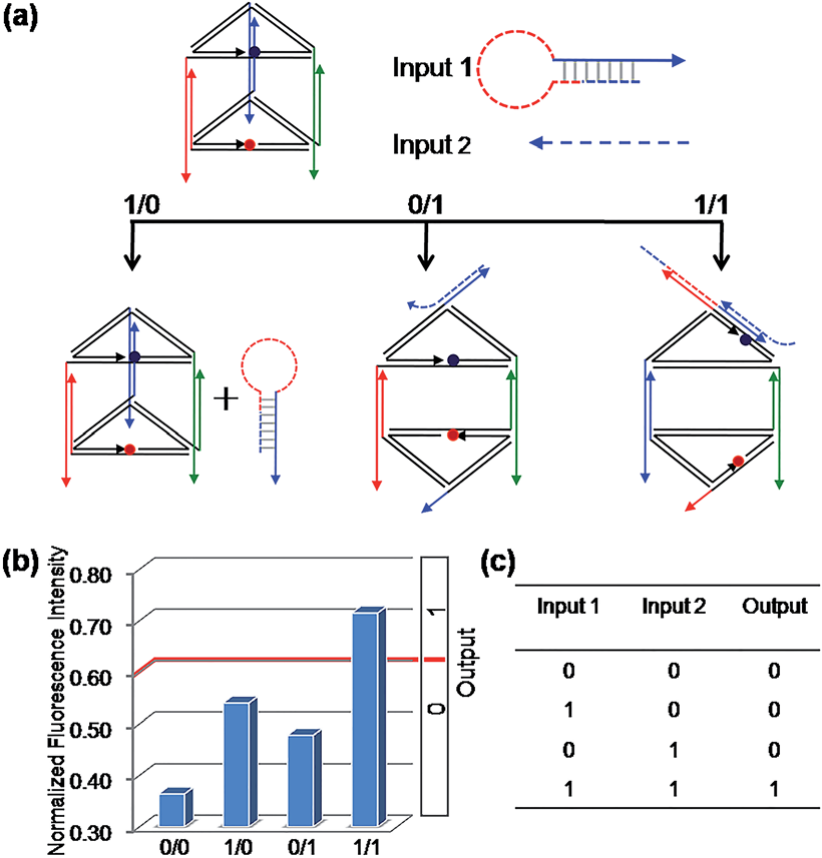
The binary “AND” logic gate. (a) Diagram of the operational design of the “AND” gate. Solid and dashed lines with the same color are complementary to each other. (b) The NFI_603_ of ROX. (c) The truth table of the “AND” logic gate.

As mentioned above, we used the same DNA nanoprism as a universal work unit throughout the research. The universality of the DNA nanoprism enables our system to be operated easily for many different kinds of molecular logic gates by simply altering the sequences of the input strands, while retaining the same DNA nanoprism as a versatile work unit. As shown in Fig. 1, though C3 is able to induce a reconfiguration of the prism, the resulting normalized fluorescence intensity value is not obviously changed and is less than the threshold value. Based on this phenomenon, the sequences of C2 and C3 were modified slightly as the respective Input 1 and Input 2 for a binary AND logic gate (Fig. 3). Input 1 contains the C2 sequence as a part of it and forms a hairpin structure (named: C2-hairpin, Scheme S2, ESI†), thus this inert Input 1 will not open the prism because of the caged C2; Input 2 includes the C3 sequence and an additional 2 nt at the 3′ end. Since Input 1 is rationally designed with the complementary sequence of Input 2 in its stem and toehold regions, Input 2 will open preferentially the hairpin structure of Input 1 *via* toehold-mediated displacement, which releases the C2 segment and consequently unfastens the edge 2 of the prism. The activation of Input 1 by Input 2 deconstructed the prism, resulting in a high fluorescent signal, while the addition of either Input 1 or Input 2 obtained a low fluorescence signal. Thus, the true output [1] was given only from the input state (1/1), but not from the input states (0/0), (1/0) and (0/1), which is consistent with the proper execution of the AND logic gate. In fact, Input 2 opening the hairpin structure of Input 1 was a YES gate, the output of this YES gate served as an effective Input 1. Hence, it has also proved that the output of one gate in this system could be used as the input for the downstream gate based on the sequence-addressability of DNA.

**Fig. 4:**
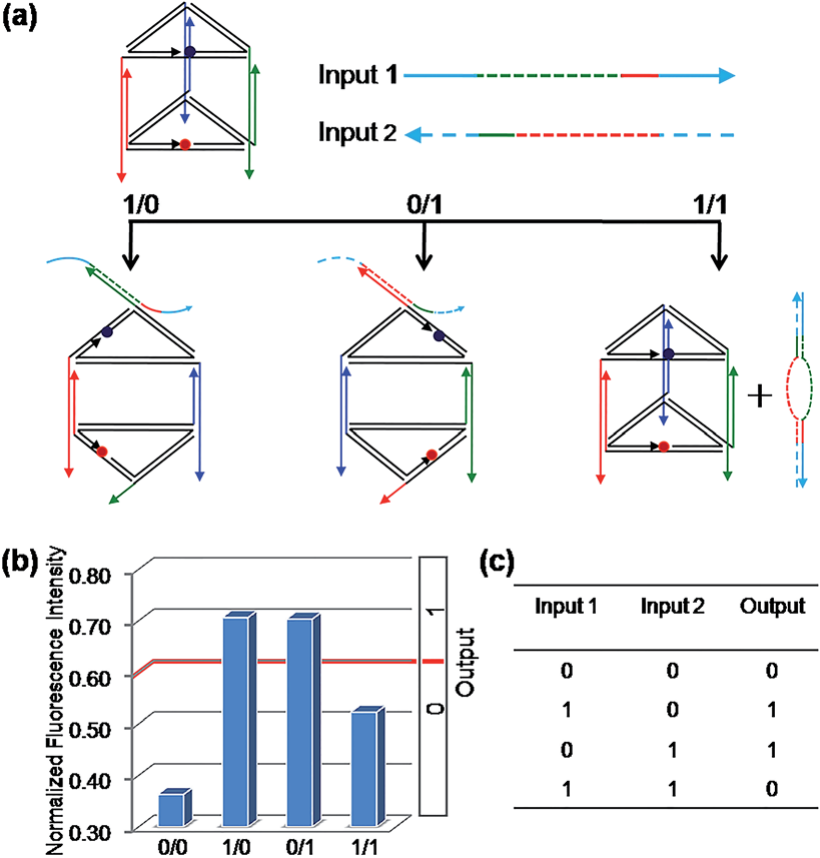
The binary “XOR” logic gate. (a) Diagram of the operational design of the “XOR” gate. Solid and dashed lines with the same color are complementary to each other. (b) The NFI_603_ of ROX. (c) The truth table of the “XOR” logic gate.

To construct a binary XOR logic gate (Fig. 4), both C1 and C2 were extended at both terminals by adding 10 and 14 nucleotides at the 5′ terminal and the 3′ terminal, respectively, which served as the respective Input 1 and Input 2. Thus, when both inputs are present simultaneously, these extended domains, which are complementary to each other, will make both inputs bind to each other preferentially instead of binding to the edges of the prism, failing to open the prism. As expected, the fluorescent signals were observed in accordance with the binary XOR gate, the true output [1] was obtained from the input states (1/0) and (0/1), while other input states produced the false output [0].

### Construction of binary combinatorial logic gates

**Fig. 5:**
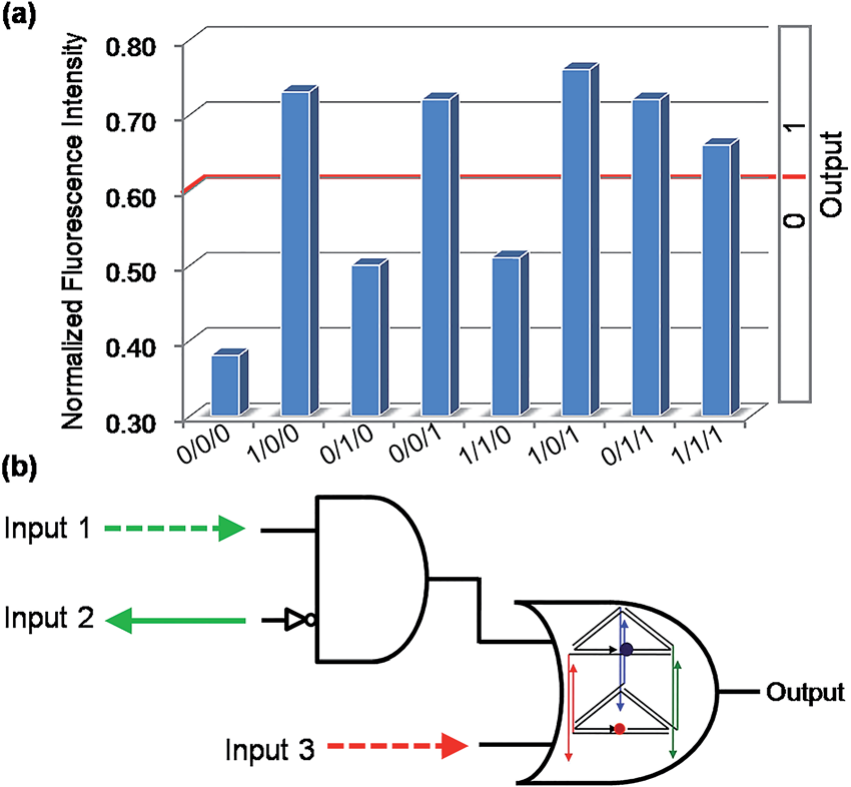
The binary three-input combinatorial gate (INHIBIT–OR). (a) The NFI_603_ of ROX. (b) Electronic equivalent circuitry.

With multiple inputs, a gate can process a higher volume of information. Though some elementary logic gates have been desirably achieved by the nanoprism, to demonstrate the gate networking necessary for the complex multi-level integrated circuits, two three-input combinatorial gates were constructed by combining an INHIBIT gate with an OR gate. For the first combinatorial gate, C1, C1′, C2, which were used in the operation of the INHIBIT gate (Fig. S6†) and OR gate (Fig. 2), were selected as Input 1, Input 2 and Input 3, respectively. As shown in Fig. 5, when the input states were (0/0/0, 0/1/0, 1/1/0), the output was [0], while the other input states (1/0/0, 0/0/1, 1/0/1, 0/1/1, 1/1/1) displayed output [1]. This is a C1 and C1′ inputed INHIBIT gate integrated with C2 into an OR gate, which is a three-input INHIBIT–OR combinatorial gate. Similarly, another combinatorial INHIBIT–OR gate was achieved by utilizing C1, C2 and C2′ as the inputs (Fig. S8, ESI†). It is notable that owing to the universality of this nanoprism and the Input/Output compatibility, these integrated gates were readily achieved without the design of any extra components, which decreases dramatically the difficulty and cost of operation. This also demonstrates the possibility of cascading elementary logic gates.

### Establishment of a logic gate system for identification of even numbers and odd numbers from natural numbers

**Fig. 6:**
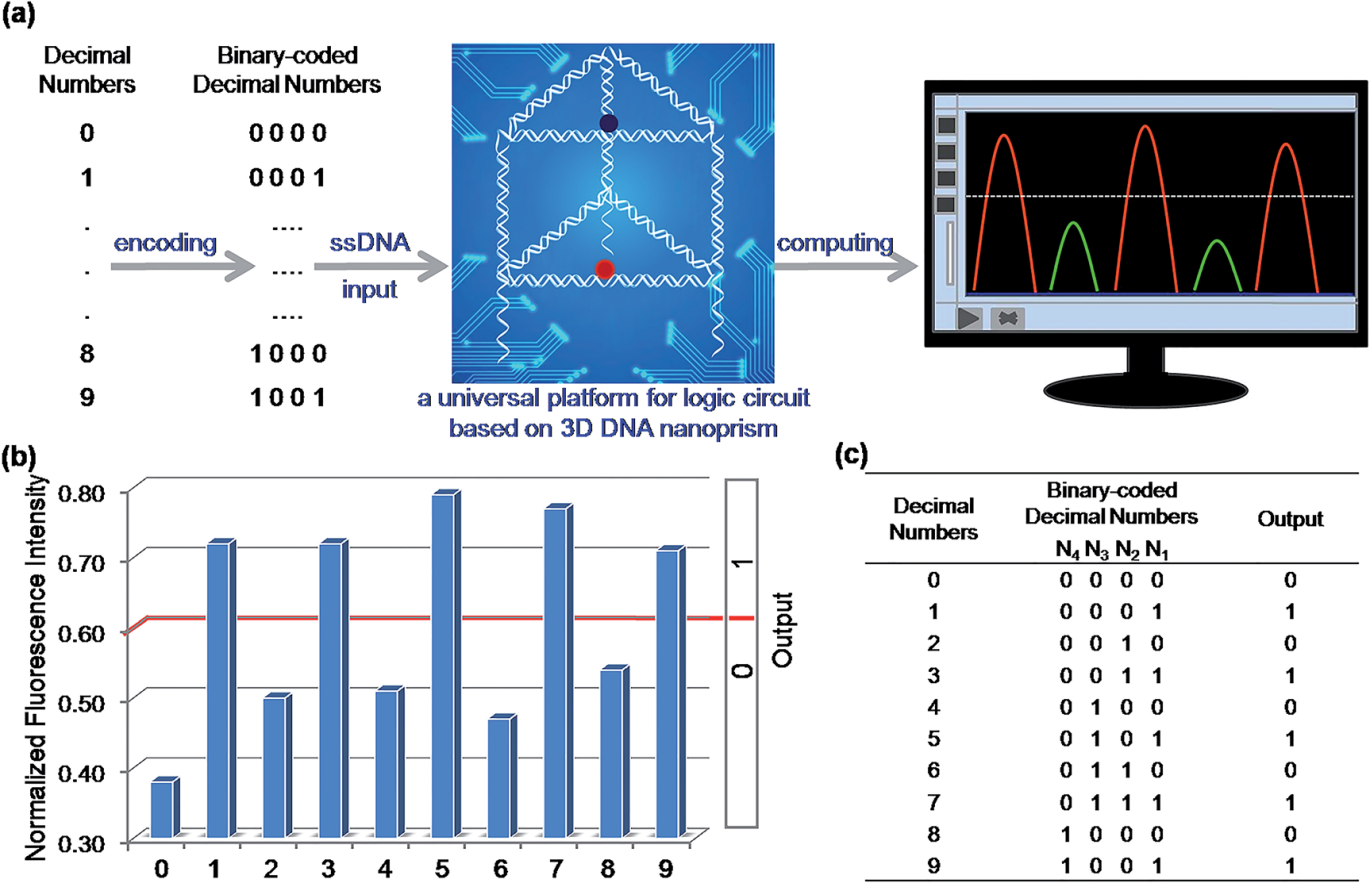
A logic gate system for identification of even numbers and odd numbers from natural numbers less than 10 by employing the DNA nanoprism as a universal platform. (a) Diagram of the operational processes of computation. (b) The NFI_603_ of ROX. (c) The truth table of computational results.

To demonstrate the application prospect of this universal DNA nanoprism platform, we applied it to solve a challenging practical problem: identification of even numbers and odd numbers from natural numbers less than 10. Firstly, these decimal numbers were encoded with binary-coded decimal (BCD) code. BCD code is a class of binary encodings widely used in financial, commercial, and industrial computing. It is more accurate when representing and rounding decimal quantities as well as easy to convert into a human-readable representation.^38^,^39^ As shown in Fig. 6(a), each decimal digit was transformed into a four-bit binary number (*N*_4_*N*_3_*N*_2_*N*_1_), then four short strands of ssDNA used in the foregoing elementary logic gates were chosen to assign to the four bits as the inputs, respectively. Here, C2-hairpin, C3, C1′ and C2, which were used in the aforementioned binary basic logic gates, were chosen and assigned to the respective bit, *N*_4_, *N*_3_, *N*_2_ and *N*_1_, for computing. A detailed schematic illustration of the computation is shown in Table S2.† The computational results of the four-input binary logic gate system are shown in Fig. 6(b) and (c). The DNA nanoprism worked perfectly; as expected, the corresponding NFI_603_ values of all the odd numbers were greater than the threshold value 0.6, presenting a true output, while the even numbers produced a false output. Since whether a natural number is either odd or even is determined by the digit in the units position, the logic system established here is able to distinguish all the odd numbers from the even numbers with a proper pretreatment, no matter whether the number is big or small. This interesting phenomenon proved that the DNA nanoprism is smart enough to respond to complex external stimuli for computation. Due to the integration and universality of this nanoprism, such complex calculations were easily accomplished by employing only this single nanoprism and four short single-stranded DNA, without designing multiple functional components laboriously.

### Construction of ternary logic gates

**Fig. 7:**
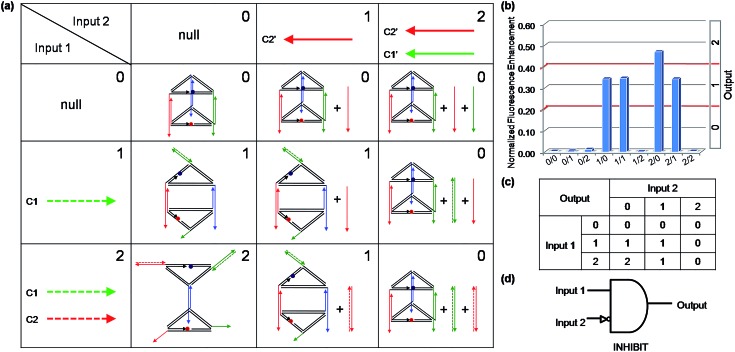
The ternary “INHIBIT” logic gate. (a) Diagram of the operational design of the ternary “INHIBIT” gate. Solid and dashed lines with the same color are complementary to each other. (b) The normalized fluorescence enhancement of ROX at 603 nm. (c) The truth table. (d) Electronic equivalent circuitry.

Binary logic is the basis of modern computers. However, logic gates will encounter uncertainty and imprecision when processing complex information. In these cases, binary logic often struggles to process information, since it has only two states, that is: on and off, or true and false. Fortunately, multi-valued logic can be constructed to make up for the imperfection of binary logic. Multi-valued logic involves switches between more than two states. Increasing the number of distinguishable states will bring about higher data storage densities and more powerful information processing capability. Recently, some multi-valued logic functions were realized,^40^,^41^ but nucleic-acid-based multi-valued logic gates remain rare, which is probably due to the fact that it is difficult to obtain a nucleic acids based nanostructure which can convert between multistable states. The 3D DNA triangular prism fabricated in this work is capable of receiving different input information to produce seven kinds of distinguishable nanostructures, which makes it an ideal candidate for multi-valued logic gate construction. To demonstrate the capability of this DNA prism in implementation of multi-valued logic gates, a ternary logic gate was realized as a case in point. The ternary logic gate, which is the best choice for practical application in multi-valued logic,^42^ is one that has two inputs which represent three states (say 0, 1 and 2) and generates one output that can have one of the three states. As an illustration, a ternary INHIBIT logic gate was operated by utilizing the same prism as the work unit. Before operating the ternary INHIBIT logic gate, we defined the two inputs and the outputs. As shown in Fig. 7(a), for Input 1, the values [1] and [2] represent the introduction of C1 and the combination of C1 and C2 with the system, respectively. For Input 2, the value [1] and [2] represent the addition of C2′ and the combination of C2′ and C1′, respectively. For the two inputs, the value [0] represents that no ssDNA was added. Then three logic outputs values [0], [1], and [2] were assigned to three different DNA nanostructures: the intact prism, the reconfigured structure mono-SD(C1), and the reconfigured structure di-SD(C1/C2), respectively. The respective fluorescence enhancements of these nanostructures compared with that of the intact prism served as the output signals. As aforementioned, C1 or C2, as well as the two strands together, can open the prism, while these reconfigurations will be prevented by the presence of their respective complementary C1′ and C2′. Thus, the different values of the two inputs interact with the DNA nanoprism to produce the different nanostructures shown in Fig. 7(a). The input states (0/0, 0/1, 0/2, 1/2, 2/2) retained the intact prism, the output was [0]; the input states (1/0, 1/1, 2/1) generated the structure mono-SD(C1), corresponding to output [1]; and the input state (2/0) produced structure di-SD(C1/C2), displaying output [2]. The read-out of the computational results is displayed in Fig. 7(b). It is worth mentioning that not only the logic gates constructed here but also other multi-valued logic gates could be created readily by introducing multiple fluorescent reporter moieties at different positions, such as the vertices of the prism.

### Switching the prism between the opened state and closed state

In a logic operating system, it is necessary to recover the initial state of the major component readily through proper manipulation, which is a challenge in the molecular computing field. Herein, we tested whether the DNA prism can be cycled between the closed state and the opened state. As shown in Fig. S9(a),† after the prism was opened by the input strand (Is), an erasing strand (Es) was applied to erase the Is from the DNA prism *via* toehold-mediated strand displacement. Specifically, Is includes a C1 or C2 sequence and an additional 10 nt toehold domain at the 5′ end. After the prism was opened by the Is, the Es, which is complementary to Is, was introduced to hybridize with Is through its toehold domain and pulled the Is off the prism, allowing the prism to return to its initial closed state. The prism was switched between the opened and closed states driven by Is and Es, which acted as the fuel of this cycle and produced the waste double-stranded DNA (Wd). The fluorescence signal of the prism was employed to record the cycling results, and this indicated that the reversible ON (high fluorescence intensity) – OFF (low fluorescence intensity) switches operated well on either vertical edge 2 (Fig. S9(b)†) or 1 (Fig. S9(c)†) by the successive addition of Is and Es. The prism could still be closed well after having been opened three times.

### Logic gates operation in biological matrix

For biomolecular logic circuits, a desirable logic operating system must be resistant to interference and able to operate stably in a biological matrix. Since all the aforementioned logic gates were implemented under ideal experimental conditions, we wondered whether this DNA nanoprism platform would continue to perform as expected in a complex environment. We employed human blood serum, a complex mixture of nucleases, proteins, electrolytes, hormones, and so on, to represent a biological matrix for investigating the operation status of this DNA nanoprism. Firstly, we inspected the stability of the nanoprism in serum, which is the prerequisite for logic operation. The fluorescence results showed that the DNA prism could keep its structure intact for about six hours in 10% (v/v) human blood serum (Fig. S10†). This is likely due to that 3D nanostructure provides its component DNA strands with better serum stability against nuclease degradation.^32^–^34^ Then, a binary OR logic gate was carried out in 10% (v/v) human blood serum as an example, since it involves most of the typical reconfigurations. As shown in Fig. S11,† the 3D DNA nanoprism worked perfectly in 10% (v/v) human blood serum as it did under ideal experimental conditions. The results are encouraging for future function tests *in vivo*, such as intelligent disease diagnostics and therapy with this DNA prism.

## Conclusions

In summary, we have integrated multiple components of a logic device into a 3D DNA triangular nanoprism and utilized DNA strand displacement to reconfigure this self-assembled nanoprism for logic gate operations. Taking the nanoprism as a versatile platform and ssDNA as inputs, we successfully established a set of binary basic logic gates (OR, AND, INHIBIT and XOR), combinatorial gates (INHIBIT–OR), and multi-valued logic gates (ternary INHIBIT gate). Moreover, a logic gate system for identification of even numbers and odd numbers from natural numbers were readily established by utilizing only this single DNA nanoprism and four short single-stranded DNA. This triangular prism, as a prototype for proof of concept, integrates multiple functional components of a logic device, which significantly simplifies the design of diverse logic gates simply by altering the input strands while retaining the same DNA nanoprism. Moreover, more complex logic operations and computations could be performed efficiently and reliably as more signal reception channels and reporter moieties are integrated into other polyhedral prisms with more faces, such as tetragonal, pentagonal, and hexagonal prisms, which are also easily fabricated as we previously demonstrated.^27^ The versatility would also benefit the modular design and inter-connectivity of functional components for DNA logic circuits. The versatility, biological stability and cooperative integration endow this DNA nanoprism with exciting prospects for the design of intelligent DNA nanodevices. A nanoscale “integrated circuit board” for molecular computation is expected to be achieved if multiple DNA nanoprisms are assembled into a flat DNA origami structure. Furthermore, assembling functional nucleic acids, such as aptamers, into the DNA nanoprism will allow it to be responsive to more complicated DNA–protein or DNA–small molecule interactions, thus making this 3D DNA nanostructure greatly promising for biological, biomedical, nano-mechanical, and bio-electronic applications, such as biocomputing, multi-parameter bio-sensing, stimuli-responsive cargo delivery, and so on.

## Experimental

### Materials and reagents

The computer program of NanoEngineer-1 (version 1.1.1, Nanorex INC.) was used to facilitate the structural design, and all DNA strands were designed by a computer program “SEQUIN” (N. C. Seeman, *J. Biomol. Struct. Dyn.* 1990, **8**, 573–581), synthesized and purified using HPLC from Sangon Biotechnology Co., Ltd. (Shanghai, China). The description and sequences of DNA strands are given in Table S1.† Fresh human blood serum samples were obtained from Hunan Provincial People's Hospital, China. All other chemicals of analytical grade were purchased from Sigma-Aldrich and were used without further purification. Ultrapure water with an electric resistance of 18.25 MΩ•cm was obtained from a Millipore filtration system and used throughout. DNA strands were dissolved in water as stock solution and quantified by the absorbance at 260 nm.

#### Instrumentations

To measure the DNA concentration, absorption intensities were recorded at *λ* 260 nm using a Beckman DU-800 spectrophotometer (USA). Fluorescence spectra were measured on a QuantaMaster™ fluorescence spectrophotometer, PTI (Canada) with excitation at 588 nm (5 nm slit width) and emission at 603 nm (5 nm slit width).

### Self-assembly of DNA triangular prism

The prism construction followed a two-step self-assembly: (1) in a 1× TAE–Mg^2+^ buffer (40 mM Tris base, pH 8.0, 20 mM acetic acid, 2 mM EDTA, and 12.5 mM Mg(CH_3_COO)_2_), the central strand L, and three short strands A1, A2 and A3 (or three short strands B1, B2 and B3) were mixed at 1 : 1 : 1 : 1 ratio and annealed using an automated PCR thermocycler for self-assembly of equilateral triangular face motif M_A_ (or motif M_B_), the anneal process was 95 °C/5 min, 65 °C/30 min, 50 °C/30 min, 37 °C/30 min, 22 °C/30 min, and 4 °C/30 min; (2) prism formation: motif M_A_ and M_B_ were mixed together at 1 : 1 ratio and then re-annealed using an automated PCR thermocycler: 50 °C/30 min, 37 °C/30 min, 22 °C/30 min, and 4 °C/30 min. The final concentration of the self-assembled DNA prism was 165 nM. The prism was stored at 4 °C in the dark as a stock solution for further use.

### General method of logic gates operation

All the logic gates were operated in a 1× TAE–Mg^2+^ buffer (40 mM Tris base, pH 8.0, 20 mM acetic acid, 2 mM EDTA, and 12.5 mM Mg(CH_3_COO)_2_). For all the binary logic gates and the computing system, the prism at a concentration of 33 nM was employed and a universal normalized fluorescence intensity value of 0.6 of ROX at 603 nm (NFI_603_ = 0.6) was set as the threshold value to define a false output [0] (NFI_603_ < 0.6) and a true output [1] (NFI_603_ > 0.6). To perform the binary logic gate operations of OR, INHIBIT, XOR and the combinatorial gates, and the ternary INHIBIT logic gates, the DNA nanoprism at a concentration of 33 nM was mixed with the respective DNA inputs at a concentration of 165 nM in 1× TAE–Mg^2+^ buffer. The solutions were then incubated at 37 °C for 1.0 h. From each of the samples, an amount of 100 μL was transferred to a cuvette for fluorescence measurement. For the binary AND gate and the logic gate system, the same procedure used with the above-mentioned gates were employed again, except that the hairpin DNA Input prepared with 1× TAE–Mg^2+^ buffer was heated to 95 °C for 5 min and then were slowly cooled to room temperature for the hairpin formation prior to use.

## Supplementary Material

Supplementary information
