# Supplementary material for: A universal platform for building molecular logic circuits based on a reconfigurable three-dimensional DNA nanostructure
Source: Chem Sci. 2015 Apr 8;6(6):3556–64. doi: 10.1039/c5sc00371g (PMC6085728; doi:10.1039/c5sc00371g)
Supplement: Supplementary file 1 [file SC-006-C5SC00371G-s001.pdf]

Electronic Supplementary Material (ESI) for Chemical Science.
This journal is © The Royal Society of Chemistry 2015

Supplementary information

A Universal Platform for Building Molecular Logic Circuit Based on a Reconfigurable Three-Dimensional DNA Nanostructure

Kaiyu He, Yong Li, Binbin Xiang, Peng Zhao, Yufang Hu, Yan Huang, Wang Li, Zhou Nie, and Shouzhuo Yao*

State Key Laboratory of Chemo/Biosensing and Chemometrics, College of Chemistry and Chemical Engineering, Hunan University, Changsha, 410082, P. R. China

* To whom correspondence should be addressed. Phone: +86-731-88821626. Fax: +86-731-88821848. E-mail: niezhou.hnu@gmail.com.

Experimental section

Native polyacrylamide gel electrophoresis (PAGE). The self-assembly of the prism was characterized by native polyacrylamidegel electrophoresis. All the samples were prepared with 1×TAE-Mg^{2+} buffer (40 mM Tris base, pH 8.0, 20 mM acetic acid, 2 mM EDTA, and 12.5 mM $Mg(CH_3COO)_2$) and then electrophoresed on a 6% polyacrylamide gel (19:1 acrylamide/bisacrylamide). The electrophoresis was carried out in 1×TAE-Mg^{2+} buffer at 4 °C for 2.5 h (85 V, constant voltage). The gel was stained with SYBR@Green II for 0.5 h, and then scanned using a Gel-Doc (Bio-Rad) digital gel documentation system. The reconfiguration results of the prism after the strand displacements by different DNA inputs were also examined through native PAGE. The prism was mixed with different combinations of C1, C2, and C3 in 1×TAE-Mg^{2+} buffer. After being incubated at 37 °C for 1.0 h, the samples were electrophoresed on a 6% polyacrylamide gel (19:1 acrylamide/bisacrylamide) in the same manner described for the above-mentioned assay.

Investigation of the thermal stability of the DNA prism. To investigate the thermal stability of the DNA prism, the fluorescence intensity at 603 nm was measured as the temperature was increased from 25 °C to 75 °C in steps of 2 °C / min, this experiment was achieved by using a QuantaMaster™ fluorescence spectrophotometer equipped with a temperature controller. Prior to this, the influences of photobleaching and temperature on the fluorescence intensity of the fluorophore ROX were examined, and the effects were negligible.

Switching the DNA prism between the closed state and the opened state. To prove the DNA prism could be reversibly switched between the opened state and the closed state, extremely small volumes of Is and Es were successively added to the reaction system containing a DNA

prism at a concentration of 33 nM. By making the final concentrations of Is and Es to be 33 nM and incubating the mixture at 37 °C for 1.0 h after every addition of Is or Es, reversible switch was achieved. After every addition of Is or Es and incubation, 100 μL of sample was transferred to a cuvette for fluorescence measurement.

Assay of the serum stability of the DNA prism. For the study on the stability of DNA prism structure in serum, the DNA prism was mixed with fresh sample of undiluted human blood serum in 1×TAE-Mg^{2+} buffer, and the final percentage of human blood serum was 10% (v/v). A control group without human blood serum was prepared with the same method except addition of serum. These samples were incubated at 37 °C. At time points of 0.0, 2.0, 4.0, 6.0, and 12.0 hr, 100 μL of samples were taken out from the two groups respectively and measured immediately. Before this, the influence of human blood serum on the fluorescence intensity of the fluorophore ROX was investigated, and no influence was observed.

Logic Gate Operation in Biological Matrix. To prove that the DNA nanoprism is able to execute logic operation steadily in biological matrix, a binary OR logic gate was carried out in 10% (v/v) human blood serum. The logic gate was operated in the same manner described for that in pure buffered solution, except in this case a fresh sample of undiluted human blood serum was added with slight mixing to make the overall % of human blood serum 10% (v/v) before incubation.

Table S1 DNA sequences used in this work.

Strand name	DNA sequence (5′ to 3′)
L-3′-ROX	AGGCACCATCGTGGAGTGTCACGGTGCATGGCTTGCGACT GCTACGGACGCTCTCTAGGTCTTGCC-ROX
L-5′-BHQ-2	BHQ-2- AGGCACCATCGTGGAGTGTCACGGTGCATGGCTTGCGACT GCTACGGACGCTCTCTAGGTCTTGCC
A1	AGCCATGCACCGTGACACTCCTGAATGCTGAC
A2	CGATGGTGCCTGGCAAGACCTTCTTCGAGATG
A3	GAGAGCGTCCGTAGCAGTCGCTAGTGAGGGTA
B1	AGCCATGCACCGTGACACTCCTGTCAGCATTCAGAACC
B2	CGATGGTGCCTGGCAAGACCTTCATCTCGAAGGATTGG
B3	GAGAGCGTCCGTAGCAGTCGCTTACCCTCACTAGCGAT
C1	GGTTCTGAATGCTGAC
C2	CCAATCCTTCGAGATG
C3	ATCGCTAGTGAGGGTA
C1′	GTCAGCATTCAGAACC
C2′	CATCTCGAAGGATTGG
Binary OR Input 1	C1
Binary OR Input 2	C2
Binary INH (1) Input 1	C1
Binary INH (1) Input 2	C1′
Binary INH (2) Input 1	C2
Binary INH (2) Input 2	C2′
Binary AND Input 1 (C2-hairpin)	***GGGTACC***AATCCTTCGAGATGA***GGTACCC***TCACTA
Binary AND Input 2	ATCGCTAGTGAGGGTACC

Binary XOR Input 1	ATGTCACGTCGGTTCTGAATGCTGACTTGGTGAGTCGTAC
Binary XOR Input 2	GTACGACTCACCAATCCTTCGAGATGAACCGACGTGACAT
Binary Combinatorial Gate (1) Input 1	C1
Binary Combinatorial Gate (1) Input 2	C1′
Binary Combinatorial Gate (1) Input 3	C2
Binary Combinatorial Gate (2) Input 1	C1
Binary Combinatorial Gate (2) Input 2	C2
Binary Combinatorial Gate (2) Input 3	C2′
Computing System B1	C2
Computing System B2	C1′
Computing System B3	C3
Computing System B4	Binary AND Input 1
Ternary INH Input 1 = 0	null
Ternary INH Input 1 = 1	C1
Ternary INH Input 1 = 2	C1 and C2
Ternary INH Input 2 = 0	null
Ternary INH Input 2 = 1	C2′
Ternary INH Input 2 = 2	C2′ and C1′
Is 1	ATGTCACGTCGGTTCTGAATGCTGACTT
Es 1	AAGTCAGCATTCAGAACCGACGTGACAT
Is 2	GTACGACTCACCAATCCTTCGAGATGAA
Es 2	TTCATCTCGAAGGATTGGTGAGTCGTAC

Results and Discussion

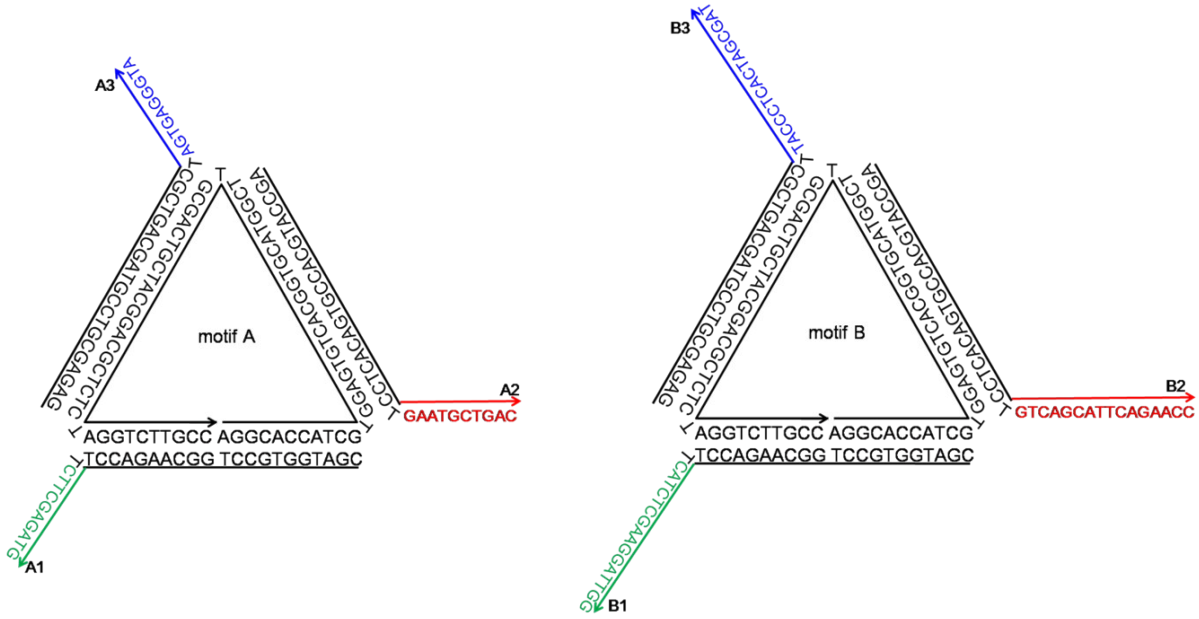

Scheme S1 The detailed distribution of DNA sequence in motif A (left) and motif B (right), respectively.

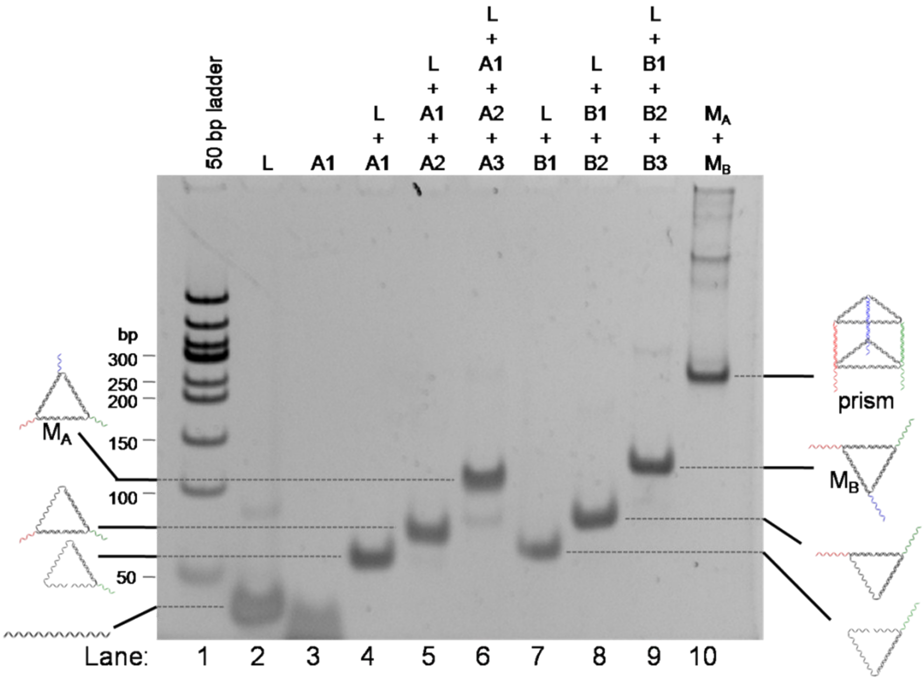

Fig. S1 Native polyacrylamide gel electrophoretic (PAGE, 6%) confirmation of DNA triangular prism self-assembly. The central strand L used in this experiment was unlabeled, and its concentration in all the assembled structures was 280 nM.

To confirm the self-assembly of the DNA triangular prism, all the well-formed DNA complexes were electrophoresed on a 6% PAGE. When the central strand L and the three short strands A1, A2 and A3 were mixed at 1:1:1:1 ratio to assemble the triangular face motif A (M_A), a major band was observed (lane 6); similarly, for the triangular face motif B (M_B), a single band appeared (lane 9) when L, B1, B2 and B3 were mixed together at 1:1:1:1 ratio for assembly. The identity of the two triangular base motifs can be confidently assigned from the purposefully prepared size marker L + A1 (1:1), L + A1+ A2 (1:1:1) and L + B1 (1:1), L + B1+ B2 (1:1:1). Upon mixing the two triangular motifs together (M_A + M_B), the bands of triangular motifs disappeared and a new dominant band appeared (lane 10), corresponding to a heterodimer of the two triangular motifs, namely the triangular prism.

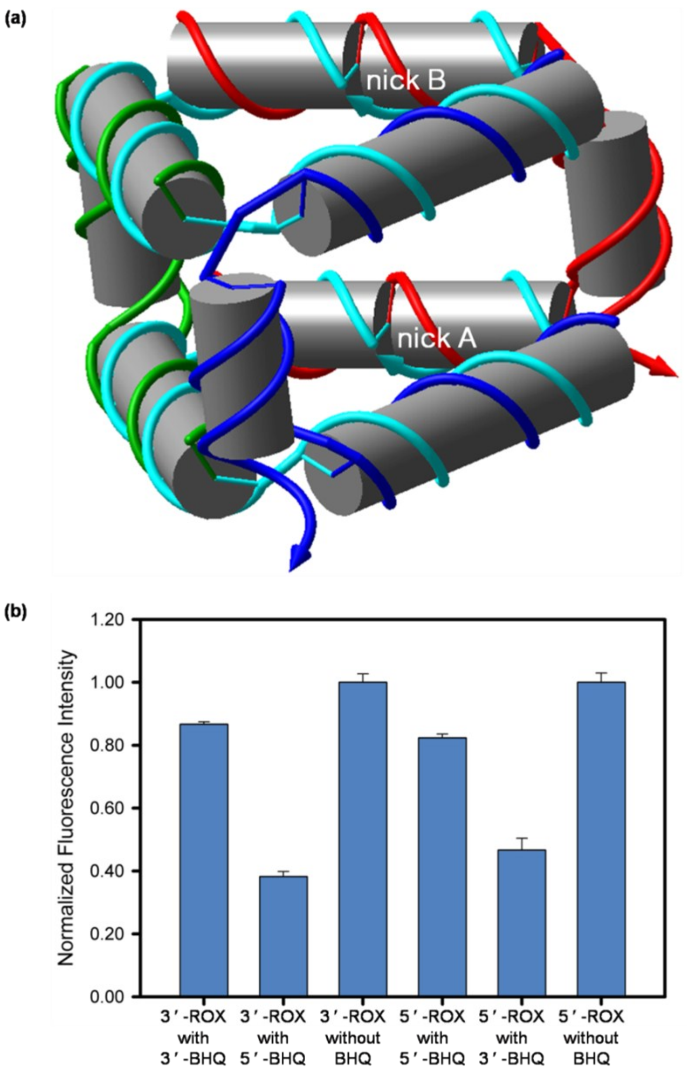

Fig. S2 The optimization of end-labeling strand L for a minimum background fluorescence of the intact prism. (a) One of the two terminals (5′ and 3′) of strand L at nick A (lower face motif) and one of the two terminals (5′ and 3′) of strand L at nick B (upper face motif) were chosen for labeling ROX and BHQ-2, respectively. (b) The normalized fluorescence intensity of ROX at 603 nm of the different combinations of labeled ROX/BHQ-2 pair. The fluorescence intensity of totally unquenched ROX in corresponding prism without BHQ-2 was normalized to 1.0 for normalization.

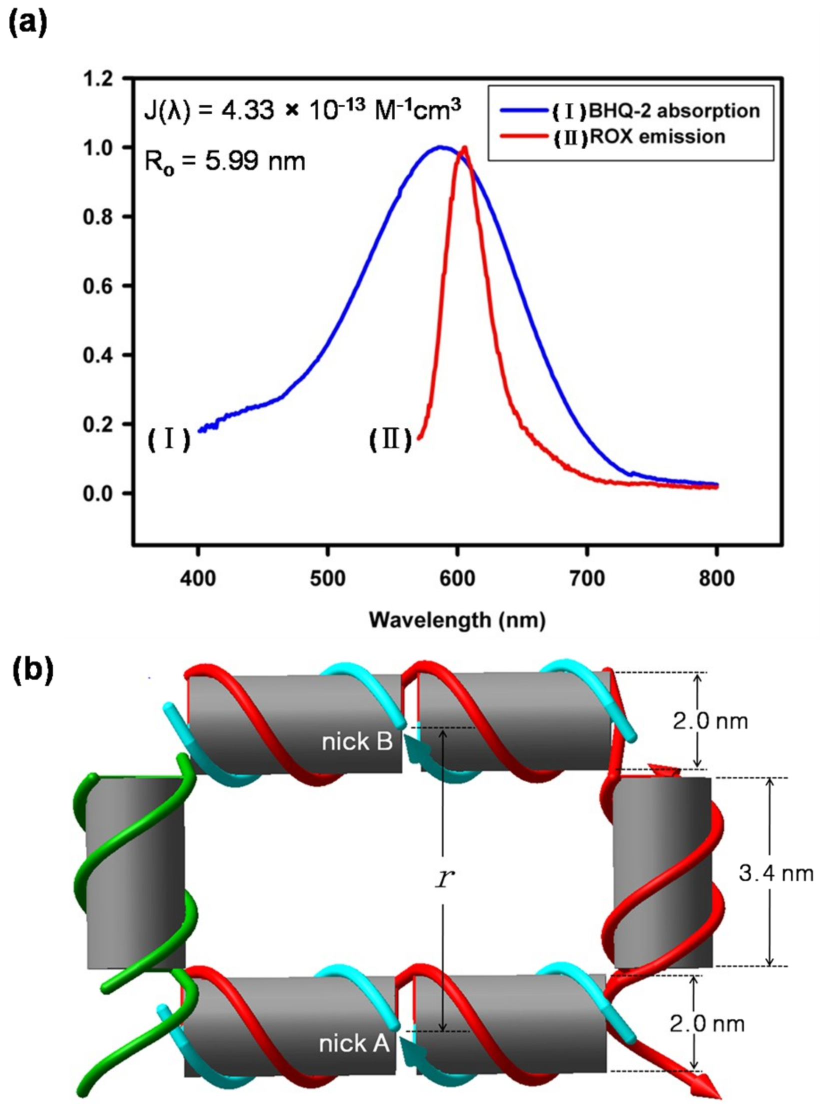

Fig. S3 Fluorescence resonance energy transfer (FRET) studies for further verification of DNA triangular prism self-assembly. (a) According to their respective fluorescence and absorption spectra, an overlap integral $J(\lambda)$ of 4.33×10^{-13} $M^{-1}cm^3$ and Förster distance R_0 of 5.99 nm were calculated for the donor/acceptor pair of ROX/BHQ-2. (b) A geometric model of part of the prism produced by the computer design program of NanoEngineer-1 (version 1.1.1, Nanorex INC.) for estimating the distance r between nick A and nick B.

The self-assembled triangular prism developed in this work has vertical sides with length about 3.4 nm (10 bp) and equilateral triangle bases with side length about 7 nm (21 bp). The size of the nanoprism perfectly matches the effective distance of FRET.[S1,S2] Thus, in this work, we

applied fluorescence resonance energy transfer (FRET) technique to indirectly prove the structure of the prism. The distance between the fluorophore and the quencher within the prism was measured by FRET study. The efficiency of energy transfer for a single donor-acceptor pair at a fixed distance is

$$E = \frac{R_0^6}{R_0^6 + r^6}$$

where r is the separation distance between donor and acceptor, R_0 is the Förster distance of this donor-acceptor pair. Given that the fluorophore ROX has a quantum yield Φ of 0.7, and according to their respective fluorescence and absorption spectra, an overlap integral $J(\lambda)$ of 4.33 × 10^{-13} $M^{-1}cm^3$ and Förster distance R_0 of 5.99 nm were calculated for the donor/acceptor pair of ROX/BHQ-2 (Fig. S3 (a)), while the maximum FRET efficiency of this donor-acceptor pair was 62.5% (the self-assembled prism fluoresces at 37.5% the unquenched fluorescence, Fig. S2), thus the distance r between ROX and BHQ-2 within the prism was calculated to be 5.5 nm. As shown in Fig. S3 (b), one helical turn of 10 bp is about 3.4 nm, and the DNA double helix diameter is about 2.0 nm, therefore the distance r between nick A and nick B in this prism with 10 bp vertical edge was estimated to be 5.4 nm (2.0 nm ÷ 2 + 3.4 nm + 2.0 nm ÷ 2) according to the geometric models in Scheme 1 and Fig. S3. The distance r between ROX and BHQ-2 is consistent well with the distance between the two nicks of the triangular motifs in this nanoprism, confirming the self-assembly of the DNA nanoprism. In our previous research to prepare the similar DNA nanoprisms with same size[S3], we have tried characterizing prisms directly by using atomic force microscope (AFM). Because the size of these triangular nanoprisms are too small, and they would probably partially collapse on the mica surface during AFM tip scanning due to all struts being single DNA duplexes, these nanoprisms appeared as

dispersed spots or blobs under AFM (data not shown). The AFM image shows little structural information about the triangular nanoprisms, and it is not a solid evidence to demonstrate the prism structures in this case. Recent research work reported by Fan's group indicated that the bigger the 3D DNA nanostructure is the more detailed structural information can be obtained by AFM.[S4] This fact enlightens us that it might be possible and easier to obtain the structural information of our nanoprisms by AFM if we prepare bigger nanoprisms with the side length of triangular bases about 3-4 turns.

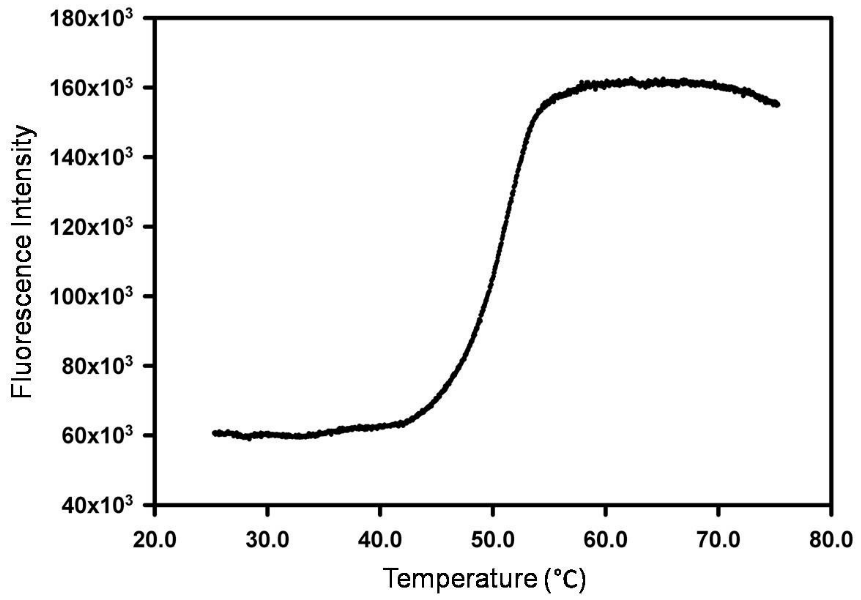

Fig. S4 The thermal stability of the DNA triangular prism structure.

The thermal stability of the triangular prism was investigated *via* the fluorescence signal change of the DNA nanoprism in response to the continuous increasing temperature (shown in Fig. S4). With the increase of temperature, the fluorescence intensity starts a sharp increase at 43 °C, suggesting that the nanoprism starts to open, and then reaches a maximum at 57 °C, indicating that two motifs are totally separate from each other. In this case, we defined a “melting point” (“Tm”) of the prism as the temperature at which the rise in the fluorescence intensity was half maximal and the “Tm” of the triangular prism was determined to be about 50 °C. Interestingly, this “Tm” value isn’t consistent with the theoretically calculated Tm values of the three vertical sides of the nanoprism, which are 35.1 °C, 33.1 °C, and 35.0 °C, respectively. There may be two reasons for this phenomenon: firstly and dominantly, since three vertical sides are connected together by two triangular bases in the prism, a synergistic effect may exist between the three vertical sides, thus the vertical duplex as the component would be more

thermally stable than it as solo duplex, which has been supported by that the temperature at which the prism starts to open its vertical side (43 °C) is remarkably higher than the Tm value of each vertical side of the nanoprism; Secondly, the increase of temperature leads to gradually open one, then two, and finally all three vertical sides, thus, at the "Tm" of the nanoprism, intact prism and different opened prisms with different fluorescent signals coexisted in the reaction system, including the mono-SD, di-SD, and tri-SD states (as shown in **Fig. 1**), which is somewhat different from the denaturation of a solo DNA duplex with only two states, the paired and the denatured. The melting curve clearly reflected the thermal stability of the prism, indicating that the prism was stable enough to be applicable at room temperature or physiological temperatures (37 °C).

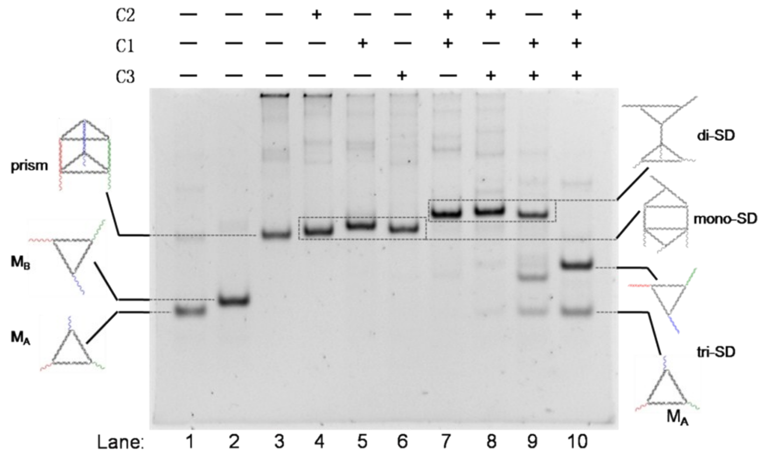

Fig. S5 Native polyacrylamide gel electrophoretic (PAGE, 6%) analysis of the reconfiguration of the DNA triangular prism after different combinations of C1, C2, and C3 were applied to initiate DNA strand displacement reactions on the prism. The central strand L used in this experiment was unlabeled, and its concentration in all the assembled structures was 330 nM.

After one of the three input strands (C1, C2 and C3) was input to initiate DNA strand displacement, similar reconfigurated nanostructures were obtained from the prism (Fig. 1 (a)). These nanostructures (lane 4, 5 and 6) migrated at a slightly slower rate than the prism (lane 3). When two of the three input strands were combined to reconfigurate the prism, nanostructures of two triangular motifs linked by a 10 bp DNA duplex were acquired (Fig. 1 (b)). These nanostructures (lane 7, 8 and 9) had similar migration rates and migrated much slower than the prism (lane 3). The nanostructure (lane 9) derived from reconfiguration induced by C1 and C3 was unstable and part of this nanostructure disassembled to generate two separate motifs probably due to the heat generation during electrophoresis. The simultaneous input of C1, C2 and C3 leaded to a deconstruction of the prism structure, producing motif M_A and motif M_B with three paired tails. These experimental results of electrophoresis were in good agreement with that of fluorescence measurements (Fig. 1), confirming the successful reconfigurations of the triangular prism.

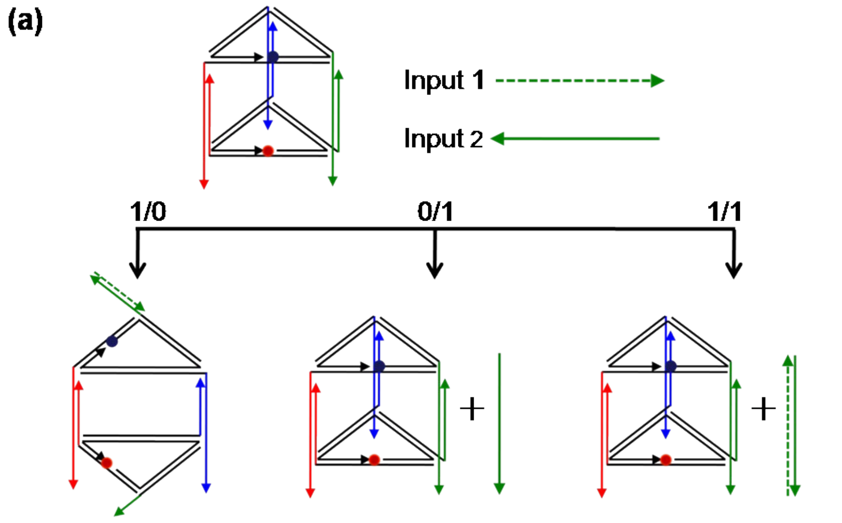

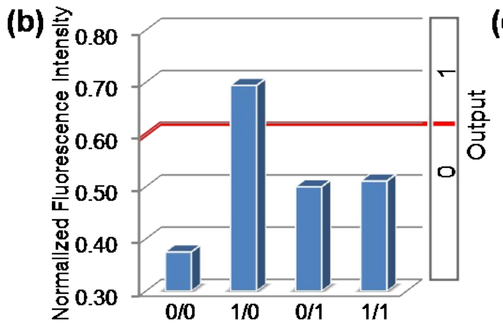

(c)

Input 1	Input 2	Output
0	0	0
1	0	1
0	1	0
1	1	0

Fig. S6 The binary "INHIBIT" logic gate *via* SDRs that occur on edge 1 of the prism. (a) Diagram of the operational design of the "INHIBIT" gate. Solid and dashed lines with the same color are complementary to each other. (b) The NFI_{603} of ROX. (c) The truth table of "INHIBIT" logic gate.

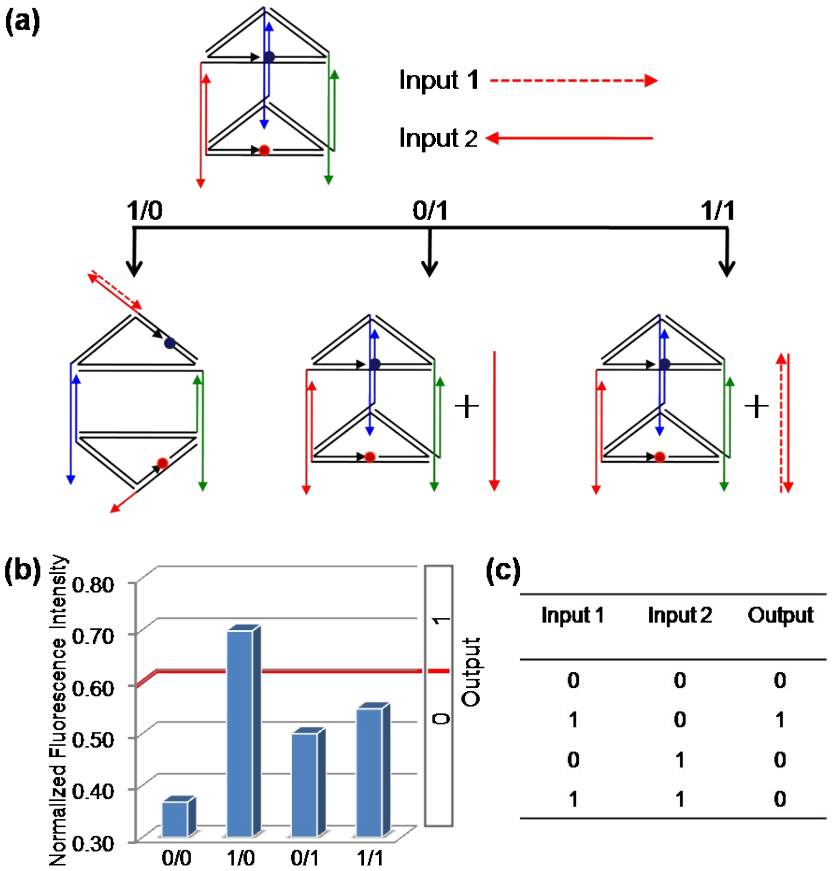

Input 1	Input 2	Output
0	0	0
1	0	1
0	1	0
1	1	0

Fig. S7 The binary "INHIBIT" logic gate *via* SDRs that occur on edge 2 of the prism. (a) Diagram of the operational design of the "INHIBIT" gate. Solid and dashed lines with the same color are complementary to each other. (b) The NFI_{603} of ROX. (c) The truth table of "INHIBIT" logic gate.

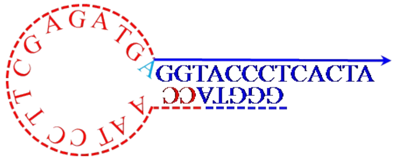

Scheme S2 The secondary structure of Input 1 of the binary "AND" logic gate under the experimental conditions. The red part is the sequence of C2. In the "AND" logic gate, Input 2 initiates a strand displacement *via* the 6-nt toehold domain at the 3′ terminal of the hairpin to open the hairpin structure of Input 1.

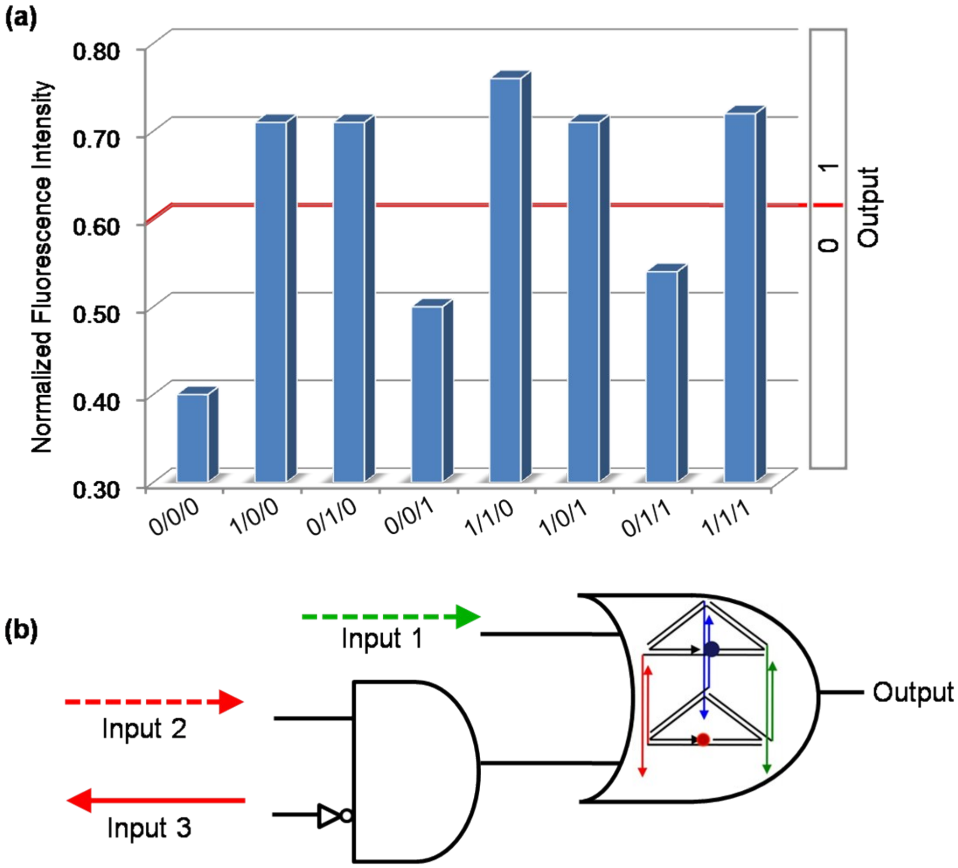

Fig. S8 The binary three-input integrated gate (INHIBIT-OR). (a) The NFI_{603} of ROX. (b) Electronic equivalent circuitry.

Table S2 Schematic illustration of distinguishing even numbers and odd numbers from natural numbers less than 10.

Decimal Numbers	Binary-coded Decimal Numbers				DNA nanoprism platform	computational results
	N_4	N_3	N_2	N_1		
	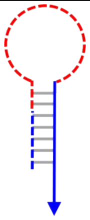	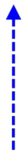	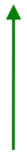	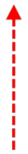		
0	0	0	0	0		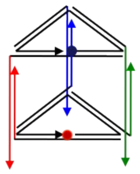<br>NFI_{603}: 0.38<br>output: [0]
1	0	0	0	1	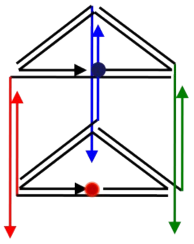	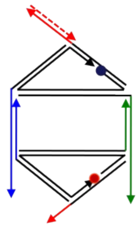<br>NFI_{603}: 0.72<br>output: [1]
2	0	0	1	0		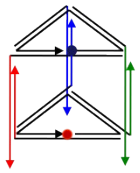<br>NFI_{603}: 0. 50<br>output: [0]

3	0	0	1	1	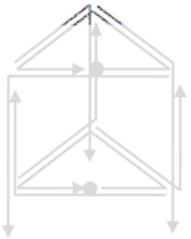	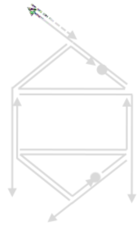<br>NFI_{603}: 0.72<br>output: [1]
4	0	1	0	0		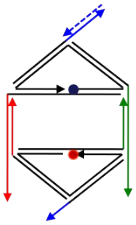<br>NFI_{603}: 0.51<br>output: [0]
5	0	1	0	1		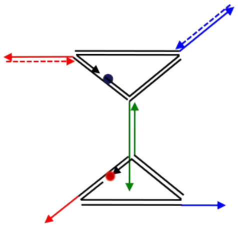<br>NFI_{603}: 0.79<br>output: [1]
6	0	1	1	0		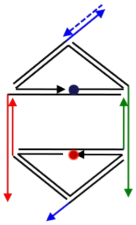<br>NFI_{603}: 0.47<br>output: [0]

7	0	1	1	1	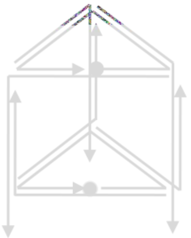	NFI_{603}: 0.77<br>output: [1]
8	1	0	0	0		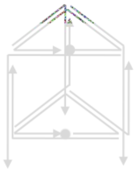 NFI_{603}: 0.54<br>output: [0]
9	1	0	0	1		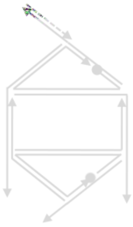 NFI_{603}: 0.71<br>output: [1]

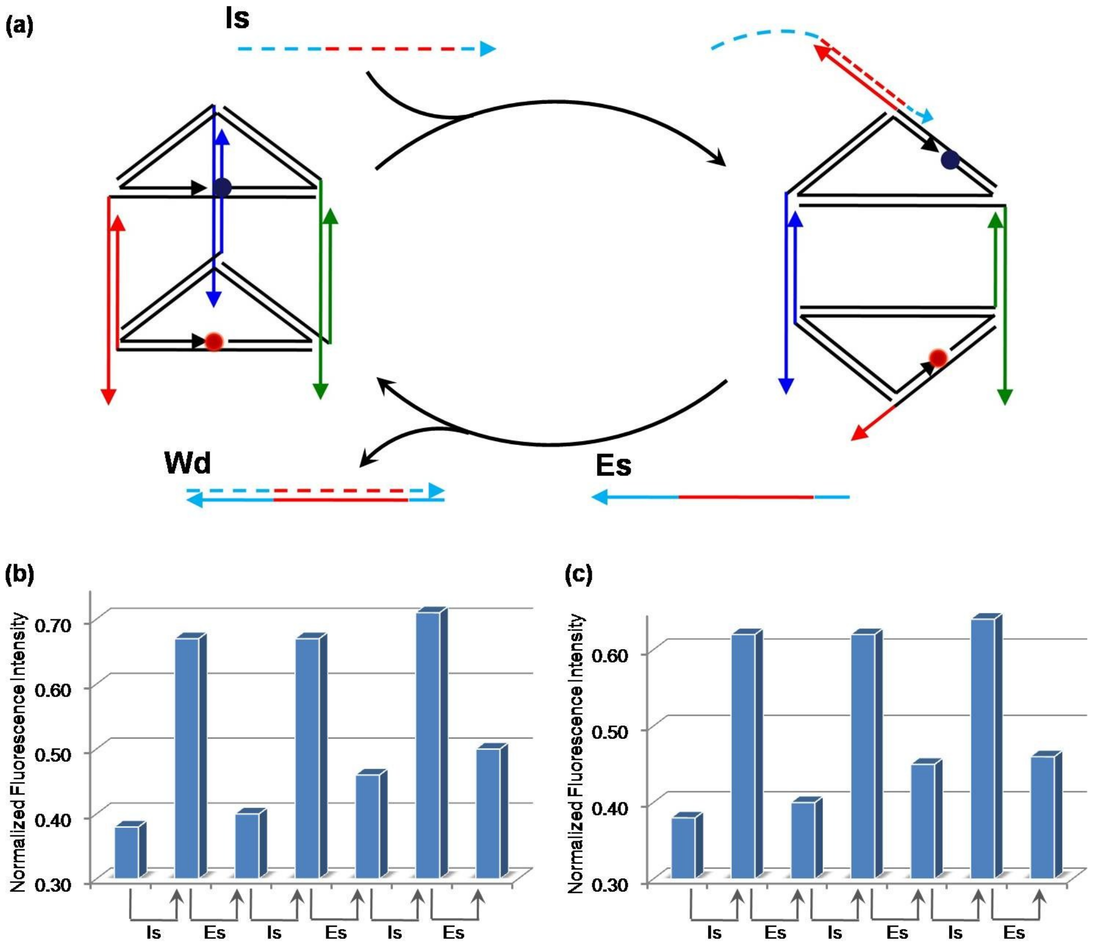

Fig. S9 (a) Scheme of the ON-OFF switch cycles between the opened state and the closed state of DNA nanoprism driven by fuel DNAs (input strand (Is) and erasing strand (Es)). Solid and dashed lines with the same color are complementary to each other. (b) The normalized fluorescence intensity of ROX at 603 nm when the cycles were operated on the vertical edge 2 (red) of the prism. (c) The normalized fluorescence intensity of ROX at 603 nm when the cycles were operated on the vertical edge 1 (green) of the prism.

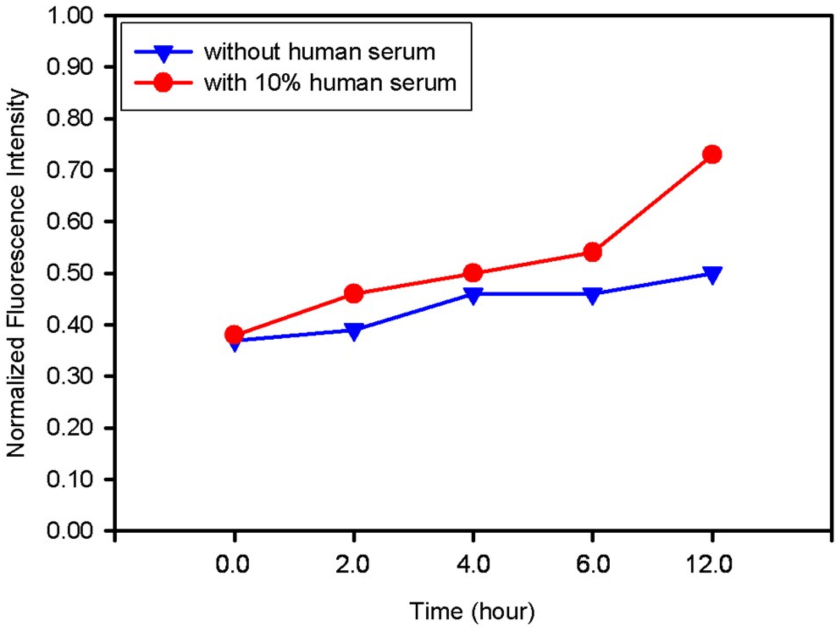

Fig. S10 The stability of the 3D DNA prism structure in serum. The results show that the DNA prism was able to keep its intact structure for about six hours in 10% (v/v) human blood serum.

For applications of the nanoprism in some specific cases, such as complex biological environments, the change of experimental conditions might make the structure of the prism unstable, for instance low concentration of cations, including Mg^{2+} or Na^{+}. In these cases, some strategies can be utilized to further enhance the stability of DNA duplex and facilitate the self-assembly of DNA nanostructures, such as small molecules that can intercalate into or complex with DNA duplex, artificial nucleic acid analogues, and modified DNA bases.[S5] Additionally, it was reported that some molecules in cells such as polyamines also have a stabilizing effect on DNA duplexes.[S6,S7] Together with the stability of 3D DNA nanostructure against nucleases degradation, these favorable factors would keep this nanoprism-based platform effective in varied user-specified situations.

(a)

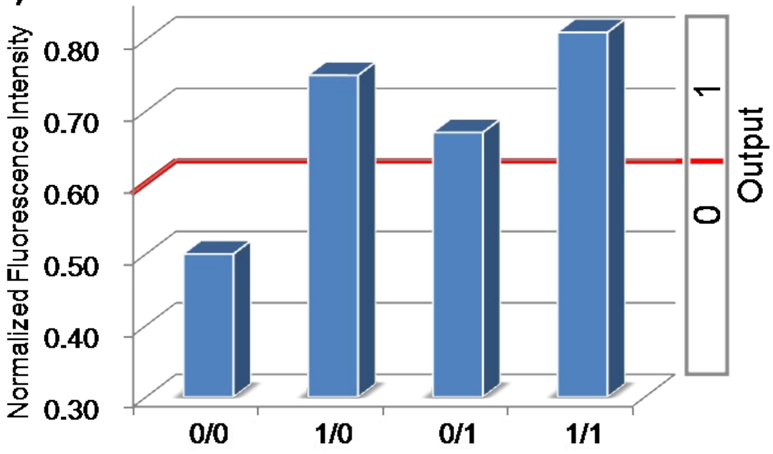

(b)

Input 1	Input 2	Output
0	0	0
1	0	1
0	1	1
1	1	1

(c)

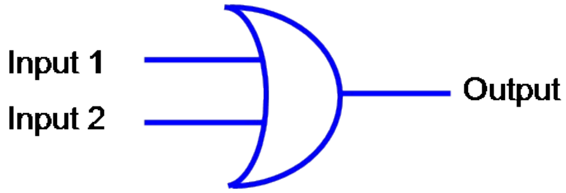

Fig. S11 A binary "OR" logic gate executed by the 3D DNA nanoprism platform in 10% (v/v) human blood serum. (a) The execution results. (b) The truth table. (c) Electronic equivalent circuitry.

References

S1 Lackowitz, J. R. in *Principles of Fluorescence Spectrosopy*; Kluwer/Plenum Publishers: New York, 1999.

S2 F. A. Aldaye and H. F. Sleiman, *J. Am. Chem. Soc.*, **2007**, *129*, 13376.

S3 Z. Nie, X. Li, Y. Li, C. Tian, P. Wang and C. Mao, *Chem. Commun.* **2013**, *49*, 2807.

S4 M. Lin, J. Wang, G. Zhou, J. Wang, N. Wu, J. Lu, J. Gao, X. Chen, J. Shi, X. Zuo, and C. Fan, *Angew. Chem. Int. Ed.* **2015**, *54,* 2151.

S5 A. A. Greschner, K. E. Bujold, and H. F. Sleiman, *J. Am. Chem. Soc.*, **2013**, *135*, 11283.

S6 B. Halle and V. P. Denisov, *Biopolymers* **1998,** *48,* 210.

S7 A. M. Distler and J. Allison, *J Am Soc Mass Spectrom* **2002**, *13*, 1129.
